# The Development of Squid Ink Melanin Nanoparticles as a Multifunctional Colorant Anchored on Hair Fibers: Preparation, Physicochemical Characterization and Dyeing Performance

**DOI:** 10.3390/biom16040573

**Published:** 2026-04-13

**Authors:** Ao Cai, Hetong Lin, Yushuang Li, Dan Li, Kaikai Bai, Junde Chen

**Affiliations:** 1 College of Food Science, Fujian Agriculture and Forestry University, Fuzhou 350002, China; 17856279701@163.com (A.C.); hetonglin@163.com (H.L.); lidan18305905087@163.com (D.L.); 2 Technical Innovation Center for Utilization of Marine Biological Resources, Third Institute of Oceanography, Ministry of Natural Resources, Xiamen 361005, China; liyushuang@tio.org.cn

**Keywords:** bionic hair colorants, squid ink melanin nanoparticles, coordination interaction, UV shielding

## Abstract

Traditional chemical hair dyes are associated with potential health risks, while botanical alternatives are often hampered by poor stability and limited color longevity. In this study, discarded squid ink was used to prepare bionic hair colorants of high performance. By synergizing ultrasound disruption with enzymatic hydrolysis, the crude ink aggregates were transformed into highly uniform squid ink melanin nanoparticles (SIMNPs) with size and zeta potential of ~174 nm and −37.5 mV, respectively. This effectively improved the solubility but reduced the steric limitation of natural melanin. To overcome the weak affinity between melanin and human hair, a biomimetic interface where Fe(III) ions act as supramolecular bridges was further engineered to stably bind the SIMNPs to hair keratin. Under optimized conditions (pH 8.0, 45 °C, and 80 min), the dyed hair achieved a natural deep black with a total color difference (ΔE*) of 68.79 ± 0.29, which was maintained at 63.19 ± 0.27 even after 13 consecutive water washing cycles. Unlike destructive oxidative dyes, this SIMNP dyeing system assisted by coordination-driven assembly preserved the native α-helical architecture and disulfide bond networks of hair keratin. Furthermore, the deposited SIMNP layer effectively protected hair fibers from ultraviolet (UV) damage due to its powerful UV-shielding capacity. Crucially, in vitro and in vivo evaluations confirmed the exceptional biosafety of this formulation, demonstrating robust cellular tolerance and absence of murine skin irritation. The work demonstrates a green, low-damage paradigm for the development of bio-based hair colorants of high performance and presents a promising pathway for the high-value utilization of marine by-products.

## 1. Introduction

As an ancient practice deeply rooted in human culture, hair dyeing has evolved into one of the most dynamic sectors of the modern cosmetic landscape, driven by escalating consumer demand and technological innovation [1]. According to recent market analysis, the global hair color market was valued at approximately USD 26.1 billion in 2024, and its value is projected to reach USD 43.34 billion by 2033 [2]. This robust trajectory underscores the immense economic potential of the sector.

Currently, the market is primarily divided into two categories: synthetic chemical dyes and natural botanical alternatives [3]. Oxidative formulations, generally based on aromatic amines like p-phenylenediamine (PPD), dominate the industry due to their low cost and superior color fastness. However, their performance comes with some health concerns [4]. Epidemiological studies have identified these synthetic precursors as potential allergens and carcinogens [5]. Research by Venkatesan et al. [6] highlighted that PPD and p-toluenediamine could induce acute toxicity and severe fiber damage, while de Souza et al. [7] warned that the oxidative intermediates of the chemical dyeing process might generate genotoxic metabolites. Beyond chemical toxicity, these harsh oxidative conditions disrupt the keratin matrix by cleaving its intrinsic disulfide bonds, causing irreversible structural damage to the hair.

Natural botanical pigments are often regarded as safer alternatives, but they have to struggle to compete with chemical dyes in terms of performance [8]. The main challenge lies in their physical and colloidal properties rather than their chemical safety. Unlike small chemical molecules, which easily diffuse into hair, natural pigments usually exist as large molecular aggregates. Due to this large particle size, they need to face nonnegligible steric hindrance, making it difficult for them to penetrate the hair cuticle [9]. They tend to be loosely adsorbed on the fiber surface of hair and fade rapidly during washing. Therefore, developing a strategy to convert the large aggregates into stable, nanoscale dispersions with high affinity for keratin might be a key to creating effective natural hair dyes.

Squid ink melanin is a type of natural eumelanin polymer composed of indole units (DHI and DHIC), known for its excellent biocompatibility [10,11], presenting great potential as a promising candidate for hair dyeing. In the squid processing industry, the ink sacs, however, are usually discarded as by-products, posing a disposal burden on the environment [12], while the valorization of this by-product into cosmetic ingredients might be of high value. The supramolecular melanin polymer component in squid ink can be utilized as a candidate black dye for hair, since cephalopods ink and human hair share similar biomolecular melanin characterized by common units like DHI and DHIC [13]. The structural homologation and high biocompatibility of squid melanin make it highly attractive to develop safe and efficient hair dye. However, like plant pigments, crude squid ink melanin suffers from poor solubility [14]. It tends to form large, insoluble clusters in water, which limits its ability to interact with hair fibers. To unlock the potential of squid ink melanin, reducing the size of melanin and improving the colloidal stability are necessary [15].

**Scheme 1:**
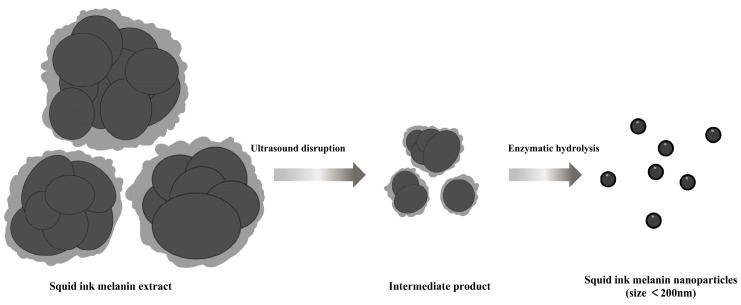
Fabrication of squid ink melanin nanoparticles (SIMNPs) by means of synergistic ultrasound disruption and enzymatic hydrolysis.

In this study, a synergistic strategy combining ultrasound disruption and enzymatic hydrolysis was employed to prepare squid ink melanin nanoparticles (SIMNPs), aiming to address issues including poor solubility and excessive particle size (see Scheme 1). Based on these SIMNPs, a novel bionic hair colorant was developed, operating through a rigorous biological mimicry. Compositionally, the colorant utilizes natural eumelanin extracted from squid ink. Because this pigment is strictly homologous to the native eumelanin found in human hair, our strategy achieves true optical biomimicry. It delivers a natural black hue that perfectly matches endogenous hair color, fundamentally differing from the harsh and artificial tones often produced by synthetic aromatic amine dyes [13]. Furthermore, rather than relying on conventional biomimetic approaches that require the harsh in situ oxidative polymerization of synthetic precursors, such as dopamine, to build artificial coatings, our strategy utilizes pre-formed SIMNPs. By employing Fe^3+^ ions to coordinate these intact nano-pigments with hair keratin, this strategy gently anchors the biomaterials onto the hair surface. This process circumvents the destructive chemical pathways of traditional oxidation. Moreover, the physicochemical properties of SIMNPs, the adsorption behavior of SIMNPs on keratin and the functional benefits (such as UV protection) of the SIMNP-dyed hair were systematically investigated, helping to reveal how melanin biopolymers interact with hair proteins at the supramolecular level [16]. Additionally, to definitively validate its potential for human cosmetic application, rigorous in vitro cytotoxicity and in vivo skin irritation assessments were comprehensively conducted. Ultimately, this bionic hair-dyeing strategy based on SIMNPs offers new insights into the design of novel hair-dyeing systems and provides a sustainable paradigm for the high-value utilization of marine by-products.

## 2. Materials and Methods

### 2.1. Materials, Cell Line and Animals

#### 2.1.1. Materials and Reagents

The squid ink sacs were obtained from Haikui Aquatic Products Group Co., Ltd. (Dongshan, China). Alkaline protease (2 × 10^5^ U/g) was purchased from Nanning Pangbo Biological Engineering Co., Ltd. (Nanning, China). L-cysteine was acquired from Shanghai Aladdin Biochemical Technology Co., Ltd. (Shanghai, China). All other chemical reagents used in this study were of analytical grade. Standardized Level 9 bleached human hair swatches, originally sourced from natural black virgin hair of Han Chinese females, were purchased from Xuchang Kousiyuan Hair Products Co., Ltd. (Xuchang, China). Prior to use, all swatches were uniformly trimmed to 20 cm. The color consistency between the original hair samples was confirmed by means of spectrophotometric measurements, and a strictly uniform baseline with minimal color variance (L* = 76.86 ± 0.41, a* = 1.59 ± 0.38, and b* = 24.72 ± 0.33) was found among all the original groups. Here L*, a* and b* represented lightness, red–green axis and yellow–blue axis, respectively [17].

#### 2.1.2. Cell Line

The human epidermal keratinocyte cell line (HaCaT; Cat No. CBP60331) was provided by Cobioer (Nanjing, China). Cells were cultured in DMEM (Yuanpei, Shanghai, China) with 10% FBS and 1% penicillin–streptomycin at 37 °C and 5% CO_2_. CCK-8 (MedChemExpress, Monmouth Junction, NJ, USA) and PBS (Servicebio, Wuhan, China) were used for cell viability assays.

#### 2.1.3. Animals

Nine female C57BL/6 mice (6–8 weeks, 20–25 g) were obtained from Xiamen Fudexin Biotechnology Co., Ltd. (Xiamen, China; License: SCXK (Min) 2025-0001) and housed in an SPF facility (22 ± 2 °C, 50 ± 10% humidity, 12 h light/dark cycle) with ad libitum access to food and water. All animal procedures were approved by the IACUC of the Third Institute of Oceanography, Ministry of Natural Resources, China (Approval No. TIO-IACUC-005, approved on 5 March 2026).

### 2.2. Preparation of Squid Ink Melanin Nanoparticles (SIMNPs)

Squid ink melanin extract (SIME) was isolated from the raw ink sacs of squid via repeated washing and centrifugation following the method by Liu et al. [18]. To prepare SIMNPs, the SIME was re-dispersed in deionized water (1:17, *w*/*v*) and processed using an ultrasonic cell probe (KQ-500B, Kunming Ultrasonic Instrument Co., Ltd., Kunming, China) at 150 W power (50% amplitude) for 1 h in pulse mode (2 s on/2 s off). After being cooled to room temperature, the pH of the dispersion was adjusted to 11.0, and the mixture was hydrolyzed by 6% alkaline protease at 50 °C for 5 h. The reaction was terminated by heat inactivation at 100 °C for 10 min. Finally, the mixture was centrifuged at 8000 rpm for 10 min to remove impurities, and the supernatant containing purified SIMNPs was collected and lyophilized.

### 2.3. Physicochemical Characterization of SIMNPs

#### 2.3.1. Transmission Electron Microscopy (TEM)

The morphology and particle size of the samples were examined using a Hitachi H-7650 TEM system (Hitachi, Tokyo, Japan) operating at an accelerating voltage of 120 kV. Prior to imaging, the samples were dispersed in deionized water, drop-cast onto a copper grid, and air-dried under ambient conditions.

#### 2.3.2. Size Distribution and Zeta Potential Analysis

The hydrodynamic diameter and zeta potential of the samples were measured using a Zetasizer instrument (Malvern Instruments, Malvern, UK). Prior to measurement, the specimens were diluted in deionized water to ensure appropriate scattering intensity and equilibrated at 25 °C. All measurements were performed in triplicate, and the results were reported as the mean value.

#### 2.3.3. Fourier Transform Infrared Spectroscopy (FT-IR)

The chemical structure and functional groups of the samples were analyzed using a VERTEX 70 spectrophotometer (Bruker, Ettlingen, Germany). The specimens were mixed with potassium bromide (KBr) powder in a ratio of 1:100 (*w*/*w*) and pressed into pellets. Subsequently, the spectra were recorded in a wavenumber range of 4000 to 400 cm^−1^ against a pure KBr background.

#### 2.3.4. UV–Visible Spectroscopy

The optical absorption profiles were recorded following the method described by Rudrappa et al. [19]. Briefly, 5 mg of the sample was dissolved in 100 mL of 2% (*w*/*v*) aqueous NaOH solution to obtain a final concentration of 0.05 mg/mL. The absorption spectra were acquired by a UV-1780 spectrophotometer (Shimadzu, Kyoto, Japan) scanning from 200 to 800 nm, using the 2% NaOH solution as the blank reference.

### 2.4. Preparation and Application of SIMNP-Based Hair Dye

#### 2.4.1. Formulation of Dye Components

To achieve optimal color depth and adhesion, a three-component dyeing system consisting of a softening agent (Agent A), a mordant (Agent B) and a colorant (Agent C), was developed based on the method by Liu et al. [17] with modifications.

Agent A (Softening Agent): This solution consisted of 5% (*w*/*v*) L-cysteine, 1% (*w*/*v*) hydroxyethyl cellulose and 0.2% (*w*/*v*) sodium dodecylbenzenesulfonate (SDBS). Its pH was adjusted to 9.0 by using ammonia solution.

Agent B (Mordant): This solution contained 4% (*w*/*v*) ferric chloride (FeCl_3_), 1% (*w*/*v*) hydroxyethyl cellulose and 0.2% (*w*/*v*) cetyltrimethylammonium bromide (CTAB). The initial 4% FeCl_3_ solution was highly acidic (measured pH = 1.86), and an ammonia solution was used to increase its pH to a milder 3.5.

Agent C (Colorant): A 5% (*w*/*v*) SIMNP suspension, utilizing the bionanoparticles prepared in Section 2.2, was mixed with 1% (*w*/*v*) hydroxyethyl cellulose to serve as the colorant. The mixture pH was adjusted to 9.0 using an ammonia solution prior to use.

The three-agent system was designed to systematically overcome the hair cuticle barrier. Firstly, Agent A acted as a softening step: ammonia (pH 9.0) swelled the cuticles and activated L-cysteine, while the anionic surfactant SDBS reduced surface tension to aid penetration. Secondly, Agent B served as the pre-mordanting step: since the 4% FeCl_3_ solution was inherently highly acidic, ammonia was utilized to adjust the pH to a milder 3.5 to prevent severe acid-induced damage to the hair keratin. Meanwhile, the cationic surfactant CTAB facilitated the wetting of the hair surface to maximize mordant deposition. Finally, Agent C delivered the colorant: ammonia (pH 9.0) ensured the optimal dispersion of SIMNPs, enabling them to coordinate effectively with the pre-anchored Fe^3+^. Throughout all three phases, the polymer hydroxyethyl cellulose acted as a crucial rheology modifier, increasing the viscosity to prevent dripping and ensuring uniform coating of the hair fibers.

#### 2.4.2. Dyeing Procedure and Color Measurement

The dyeing process involved sequentially immersing gray human hair tresses into the three agents mentioned above under controlled conditions. Specifically, the hair samples were treated with Agent A at 50 °C for 30 min, followed by Agent B at the same temperature for 30 min, and finally Agent C at 50 °C for 60 min. At the end of the dyeing process, the samples were rinsed thoroughly with deionized water and air-dried.

The dyeing effect was evaluated by measuring the CIELab color parameters (L*, a* and b*), using a spectrophotometer (SC-660A, Caipu Technology Co., Ltd., Hangzhou, China). The total color difference (ΔE*) was calculated according to Equation (1) [17], where a larger ΔE* value indicated a more distinct chromatic shift relative to the control:

(1) $${\Delta E}^{*}=\sqrt{{\left(L_{t}^{*}-L_{0}^{*}\right)}^{2}+{\left(a_{t}^{*}-a_{0}^{*}\right)}^{2}+{\left(b_{t}^{*}-b_{0}^{*}\right)}^{2}}$$

where the subscript “t” refers to the dyed sample and “0” refers to the undyed control.

#### 2.4.3. Screening of the Optimal Process Conditions for Hair Dyeing Based on SIMNPs

1. Optimization of hair-dyeing method

The influence of the mordant’s configuration on chromatic performance was investigated by comparing four distinct experimental procedures: direct dyeing (mordant-free), simultaneous mordanting, pre-mordanting, and post-mordanting. To ensure experimental rigor, all hair tresses were processed in accordance with a standardized protocol to maintain comparability. ΔE* was used as the primary metric to determine the optimal dyeing strategy.

2. Single-factor optimization of dyeing conditions

Following the establishment of the optimal mordanting sequence, the physicochemical parameters governing the performance of the colorant (Agent C) were further optimized by means of a single-factor experimental design. ΔE* was employed as the evaluation metric. The investigation systematically examined the effects of dyeing temperature (30, 35, 40, 45, 50 and 55 °C), pH value (7.5, 8.0, 8.5, 9.0, 9.5 and 10.0), and dyeing duration (20, 40, 60, 80 and 100 min) on dyeing efficiency.

### 2.5. Evaluation of the Performance of Melanin-Based Hair Dye

#### 2.5.1. Evaluation of Wash Fastness

Color durability was assessed over thirteen consecutive washing cycles following the method described by Luo et al. [20]. Each cycle comprised rinsing with tap water, gentle blotting, and thermal drying. The color difference (ΔE*) [20] was spectrophotometrically quantified after each cycle to monitor pigment leaching kinetics.

#### 2.5.2. Evaluation of Mechanical Properties

Single-fiber tensile properties were evaluated to ascertain structural resilience as previously reported [21]. Ten randomly excised filaments per group (gauge length: 30 mm) were tested using an electronic strength tester (Model XQ-1C, Shanghai Xinxian Instrument Co., Ltd., Shanghai, China). Mechanical behavior was characterized by the stress–strain profiles until fracture.

#### 2.5.3. Differential Scanning Calorimetry (DSC) Analysis

Thermal analysis was performed by using a Netzsch DSC 204F1 instrument (Netzsch, Selb, Germany), in accordance with the method described by Da Gama et al. [22]. Finely minced hair samples (approximately 2 mg) were sealed in aluminum crucibles and heated from 20 °C to 260 °C at a rate of 10 °C/min under a nitrogen flow of 100 mL/min. All thermograms were baseline-corrected against an empty crucible as a control reference.

#### 2.5.4. Evaluation of UV Resistance

Photoprotective efficacy was evaluated as described in a previous study [23] with little modification. Three experimental groups were subject to UV irradiation: undyed hair (control), commercial oxidative dye-treated hair (positive control), and SIMNP-dyed hair (test group). Samples were exposed to continuous UV irradiation by using a UV device (AHD500W, Anhongda Optoelectronics, Shenzhen, China), reaching a cumulative dose of 300 J/m^2^ over a 72 h period. This high dose was selected to effectively induce observable structural degradation in the highly keratinized hair fibers, referencing the established extreme-stress photodamage models applied in accelerated weathering tests [24]. Specifically, the 72 h exposure was designed to simulate long-term, chronic environmental photo-degradation, while the 12 h interval served as an intermediate monitoring point to capture the early onset of cuticular damage.

#### 2.5.5. Scanning Electron Microscopy (SEM) Observation

Surface topography and cuticle integrity were examined via SEM (Hitachi S-4800, Tokyo, Japan). Samples were sputter-coated with gold prior to imaging to assess architectural alignment and scale delamination [25].

### 2.6. Evaluation of Biosafety

#### 2.6.1. In Vitro Cytocompatibility Assessment

The in vitro cytocompatibility assessment was performed according to the method described by Liu et al. [26] with slight modifications. The cytocompatibility of SIMNPs was evaluated using human epidermal keratinocytes (HaCaT). Cells were seeded in 96-well culture plates at a density of 2 × 10^4^ cells/well and incubated at 37 °C with 5% CO_2_ for 24 h. Subsequently, the culture medium was replaced with complete media containing SIMNPs at various concentrations (3.125, 6.25, 12.5, 25, 50, and 100 μg/mL). After 24 h of co-incubation, cell viability was determined by means of the Cell Counting Kit-8 (CCK-8) assay by measuring the absorbance at 450 nm with a microplate reader.

#### 2.6.2. In Vivo Murine Skin Irritation Evaluation

The in vivo murine skin irritation evaluation was adapted from the protocol proposed by Seo et al. [27] with slight modifications. Briefly, 0.5 g of the commercial oxidative dye or the formulated SIMNP mixture was applied to sterile double-layer gauze (2 cm × 2 cm). The gauze was attached to the depilated dorsal region of the mice for 30 min, followed by rinsing with tap water (25 °C). A negative control group was treated with sterile physiological saline. The gauze was secured with non-irritating medical tape and a breathable bandage. This application procedure was repeated daily for 3 consecutive days. Macroscopic observations and photographs of the dorsal skin were recorded 24, 48, and 72 h after administration to monitor any occurrence of erythema or scabbing. These skin reactions were graded using the Draize scoring system [28], as shown in Table 1.

### 2.7. Statistical Analysis

All experiments were conducted with triplicate replicates per group. The results are presented as means ± standard deviations. Statistical analysis was performed by utilizing IBM SPSS Statistics 27 (IBM Corporation, Armonk, NY, USA) software. Based on one-way analysis of variance (ANOVA), Tukey’s B and Waller–Duncan tests were performed, and *p* < 0.05 was deemed statistically significant. All graphs were plotted by using Origin 2021 (Origin Lab Corporation, Northampton, MA, USA) software.

## 3. Results

### 3.1. Structural Properties of SIMNPs

#### 3.1.1. TEM Analysis of SIMNPs

**Figure 1:**
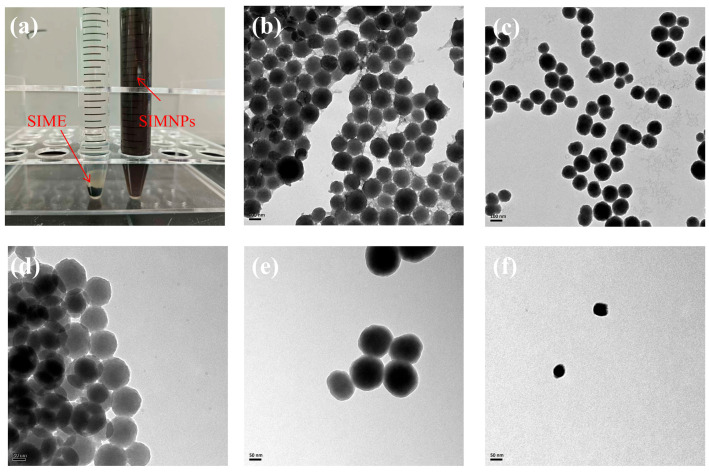
The suspension profile and transmission electron microscopy (TEM) images of SIME and SIMNPs: (**a**) SIME (**left**) and SIMNP (**right**) solutions that had been left to stand for 48 h; (**b**) SIME at 50,000× magnification; (**c**) SIMNPs at 50,000× magnification; (**d**) SIME at 100,000× magnification; (**e**) SIME after ultrasonic treatment, at 100,000× magnification; and (**f**) SIMNPs at 100,000× magnification.

After the ultrasonic treatment and enzymatic hydrolysis, the bulk melanin in the squid ink sacs was decomposed into nano-sized particles. The resultant SIMNPs were able to maintain the dispersed state for 48 h, while the bulk precursor aggregated and precipitated quickly on the bottom (Figure 1a). The microstructure and morphology of the samples were elucidated by means of TEM (Figure 1b–f). As illustrated in Figure 1b, the SIME obtained through the conventional aqueous extraction method exhibited the morphology characterized by significant aggregation, irregular compactness and indistinct boundaries. The samples featured large and irregularly shaped clusters. In sharp contrast, the SIMNPs prepared via the synergistic cell disruption–enzymatic hydrolysis strategy underwent dramatic morphological transformation (Figure 1c). The treatment effectively dismantled the bulk aggregates, yielding a population of discrete and well-dispersed bionanoparticles (Figure 1d–f). Notably, while maintaining their intrinsic quasi-spherical geometry, the SIMNPs exhibited a marked increase in roughness of the surface texture compared with the crude SIME (Figure 1f).

#### 3.1.2. Size Distribution of SIMNPs

**Figure 2:**
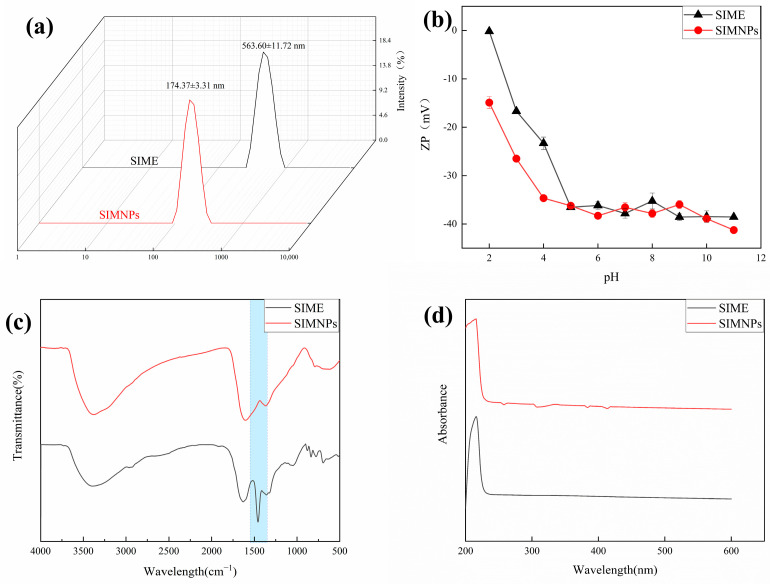
Characterization of SIME and SIMNPs. (**a**) Size distribution. (**b**) Zeta potentials measured at different pH. (**c**) Fourier transform infrared (FT-IR) spectra. The blue highlighted region (1400–1600 cm^−1^) corresponds to the characteristic absorption bands of melanin. (**d**) UV–Vis absorption spectra.

The SIME displayed an average hydrodynamic diameter of 563.60 ± 11.72 nm, coupled with a broad size distribution (Figure 2a). This large particle size and broad distribution indicated that the conventional water extraction method failed to dissociate the inherent melanoprotein complexes, resulting in the persistence of macroscopic aggregates. On the contrary, the SIMNPs exhibited a smaller mean size of 174.37 ± 3.31 nm with narrowed polydispersity (Figure 2a). This demonstrated that the synergistic treatment successfully reduced the particle size and improved the uniformity of the melanin extracts.

#### 3.1.3. Zeta Potential of SIMNPs

The surface electrochemistry of the samples was assessed by measuring the zeta potential across a broad pH range (Figure 2b). The profiles revealed distinct differences in the surface charge behavior between SIME and SIMNPs, particularly in the acidic region. The SIME exhibited an isoelectric point (IEP) near pH 2.0 (approaching 0 mV), suggesting the neutralization of surface charges at this pH. Conversely, the SIMNPs maintained a robust negative potential of approximately −16 mV even at pH 2.0, implying a lower IEP compared with SIME. Furthermore, the SIMNPs exhibited stronger electronegativity within the pH range of 2 to 5. For instance, the potential of SIMNPs reached −34.2 mV at pH 4.0, while a zeta potential of only −23.5 mV was observed for SIME. At pH 8.0, the SIMNPs demonstrated a zeta potential of approximately −37.5 mV, which was able to guarantee the stability of the nanoparticles during the dyeing process.

#### 3.1.4. FT-IR Spectra of SIMNPs

Both SIME and SIMNPs samples exhibited the characteristic absorption signature of eumelanin (Figure 2c). Specifically, the broad band at around 3400 cm^−1^ (O-H and N-H stretching) and the prominent peak at nearly 1600 cm^−1^ (ascribed to aromatic skeletal C=C and C=O vibrations) were observed. These key features confirmed the pigment’s fundamental polyindole–quinone backbone was safely preserved during enzymatic extraction. Beyond these similarities, significant spectral differences were also found. The crude SIME spectra displayed distinct peaks in the 2920–2850 cm^−1^ region and a cluttered profile in the 1000–1400 cm^−1^ “fingerprint region”. In contrast, impurity signals were not found in the FT-IR spectra of SIMNPs, and a much smoother profile overall was observed.

#### 3.1.5. UV–Vis Spectral Characterization of SIMNPs

The UV–Vis spectra of SIMNPs displayed a characteristic absorption profile with a prominent maximum at 216 nm (Figure 2d). Notably, no absorption peaks were observed at 260 nm or 280 nm for the SIMNPs, confirmed the effective enzymatic removal of nucleic acids and proteins [29]. In contrast to the crude SIME’s UV–Vis spectra showing irregular spectral fluctuations, the SIMNPs exhibited a smooth and asymptotic curve, indicating a high degree of homogeneity in nanoparticle dispersion. This result strongly corroborated the FT-IR results, specifically the disappearance of aliphatic impurity signals at 2900 cm^−1^. Both spectral techniques might validate that the refined process based on hydrolysis technology can significantly enhance both the chemical purity and structural uniformity of the nano-sized product.

### 3.2. Screening of Optimal Dyeing Conditions

**Figure 3:**
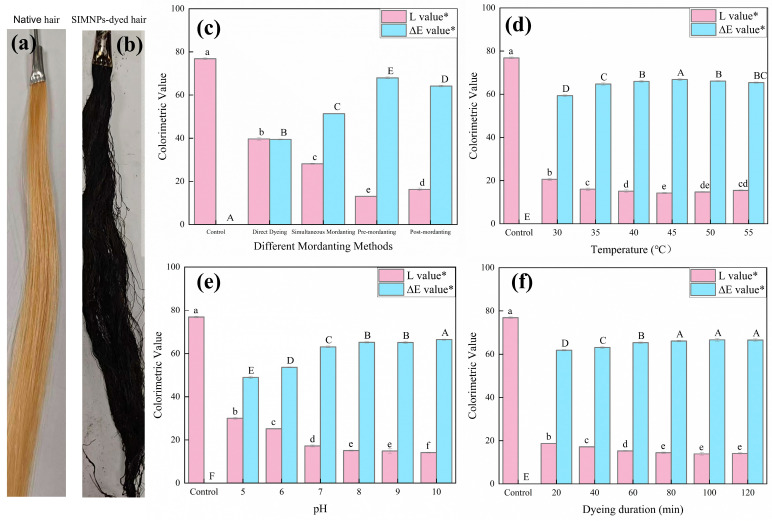
Appearance of hair dyed by SIMNPs and the optimization of the dyeing conditions. (**a**) Photograph of gray hair as control. (**b**) Photograph of hair dyed by SIMNPs. (**c**–**f**) Effects of processing parameters on the lightness (L*) and color difference (ΔE*) values of the dyed hair: (**c**) mordanting methods, (**d**) dyeing temperature, (**e**) dyeing pH and (**f**) dyeing duration. Different lowercase letters (a–f) indicate significant differences in L* values among the samples, while different uppercase letters (A–F) indicate significant differences in ΔE* values among the samples.

The hair before or after the dyeing treatment was monitored (Figure 3a,b). Bleached hair swatches with comparable color parameters (L*, a*, and b*) were randomly assigned to the corresponding experimental groups. Subsequently, the impacts of mordanting strategy, temperature, pH and duration on the colorimetric parameters (L*, a*, b*, and ΔE*) were systematically evaluated to maximize the dyeing efficacy of SIMNPs on hair (Figure 3c–f).

#### 3.2.1. Mordanting Strategy

The specific mordanting strategy exerted a decisive influence on pigment uptake. The direct dyeing method (without mordant) resulted in an excessively high luminosity value (L*), indicating negligible coloration. It confirmed the necessity of metal coordination. Among the tested protocols, the lightness values (L*) followed a descending order: direct dyeing > simultaneous mordanting > post-mordanting > pre-mordanting. Pre-mordanting yielded the deepest black shade with the lowest L* and the highest ΔE*; it was thus established as the optimal protocol (Figure 3c).

#### 3.2.2. Temperature

When evaluating the range of 30 to 50 °C, the L* value exhibited a non-monotonic trend. Among the temperatures, the best dyeing performance was found at 45 °C, where L* reached its minimum and ΔE* peaked (Figure 3d). Nonetheless, temperatures exceeding 45 °C led to a decline in color intensity. Thus, 45 °C was selected.

#### 3.2.3. pH

The L* value displayed a U-shaped profile with the increase in pH (Figure 3e). Alkaline conditions (pH 8–10) significantly enhanced dyeing efficacy, helpful to enhancing the deep black of hair. However, alkaline conditions might also induce the swelling of cuticular scales and reduce steric hindrance within hair fibers. Thus, pH 8.0 was selected to balance color depth and fiber structural integrity.

#### 3.2.4. Duration

The hairs could be quickly dyed by the SIMNP dyeing system, and significant improvement in ΔE* was observed after a 20 min dyeing process, as presented in Figure 3f. The color deepened progressively until reaching a saturation plateau at 80 min (L* minimum of 13.89 ± 0.66), indicating dynamic equilibrium.

#### 3.2.5. Validation of Optimized Protocol

Based on the single-factor experiments above, the optimal dyeing conditions were established as: pre-mordanting with Fe^3+^, pH of 8.0, 45 °C and 80 min. Confirmatory experiments under these synergistic conditions prepared hair samples with a deep natural black color (L* = 12.26 ± 0.27, a* = 0.56 ± 0.12, and b* = 1.12 ± 0.10) and a total color difference (ΔE*) of 68.79 ± 0.29. This value exceeded the maximums recorded in individual single-factor tests, confirming the validity of optimization.

### 3.3. Dyeing Performance of SIMNP-Based Hair Dye

#### 3.3.1. SEM Analysis of Hair Dyed with SIMNPs

**Figure 4:**
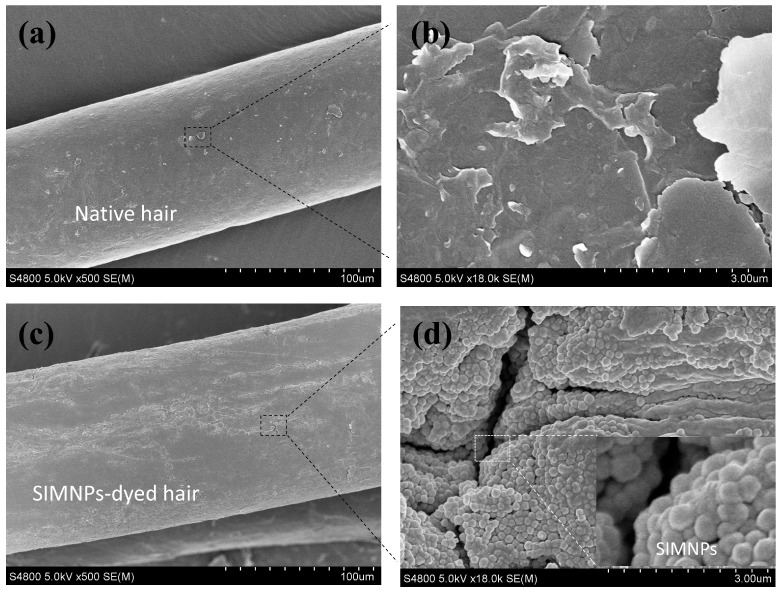
Microstructure characterization and performance evaluation of dyed hair: (**a**) SEM image of native control hair at 500× magnification. (**b**) Magnification of the selected field marked in panel a at 18,000× magnification. (**c**) SEM image of SIMNP-dyed hair at 500× magnification. (**d**) Magnification of the selected field marked in panel c at 18,000× magnification.

The surface topography of hair fibers was scrutinized via SEM to assess the effect of the dyeing process on hair. The native control hair exhibited a smooth and pristine surface with tightly imbricated cuticle scales (Figure 4a), while the inter-scale gaps of the native hair appeared clear and empty at high magnification (Figure 4b). Notably, the hair treated with the SIMNP-based dye retained comparable surface quality, evidenced by the intact cuticle layer with no observable lifting, erosion, or widening of inter-scale gaps (Figure 4c). Furthermore, unlike the control fibers shown in Figure 4b, it was found that a great number of SIMNPs were located in the gaps of hair cuticles, confirming the effective anchoring of SIMNPs on the hair fibers (Figure 4d).

#### 3.3.2. Washing Fastness

**Figure 5:**
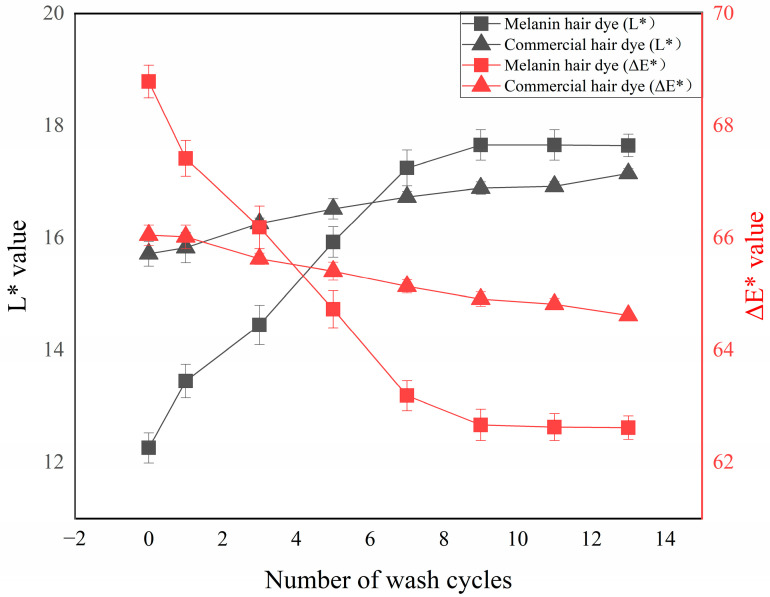
Change in color parameters (L* and ΔE*) of dyed hair during water washing (SIMNPs vs. commercial dye). Squares (■) represent the melanin hair dye, and triangles (▲) represent the commercial hair dye. Additionally, black markers indicate the L* values, while red markers indicate the ΔE* values.

The color durability was spectrophotometrically assessed over thirteen wash cycles. Commercial oxidative hair dye (PPD-based) was used as a reference standard, and this PPD-based dye demonstrated high retention with minor chromatic shifts (L* shifted from 15.72 ± 0.22 to 17.15 ± 0.09; ΔE* from 66.02 ± 0.21 to 64.62 ± 0.05). In contrast, the melanin-dyed hair exhibited a distinct biphasic washing profile (Figure 5). An initial phase of pigment leaching was observed during the first seven cycles, characterized by a rise in luminosity (L* increased from 13.45 ± 0.30 to 17.25 ± 0.32) and a decrease in color difference (ΔE* declined from 68.79 ± 0.29 to 63.19 ± 0.27). As washing continued (cycles 9–13), the chromatic values became stable, with an L* ≈ 17.25 and a ΔE* ≈ 62.85. Although the tolerance of SIMNP-based dye to water washing might be weaker than that of the PPD-based dye, the color durability of the SIMNP dyeing system was still outstanding.

#### 3.3.3. Mechanical Properties

**Figure 6:**
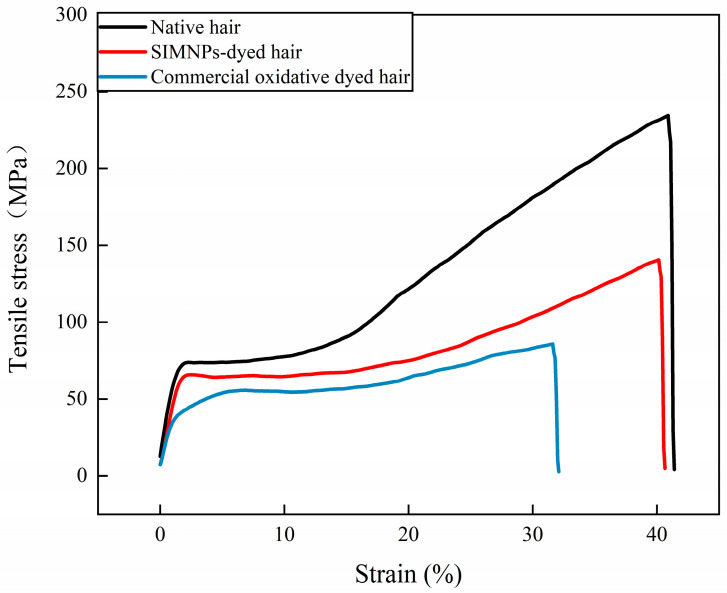
Tensile stress vs. strain curves of hair treated with different dyes. The black line represents native hair, the red line denotes SIMNP-dyed hair, and the blue line indicates commercial oxidative-dyed hair.

Tensile competence serves as a definitive biomarker for fiber health, intrinsically reflecting the stability of the α-keratin helical network and the density of the disulfide cross-links within hair fibers [30]. As shown in Figure 6, the strain test was performed to capture the impacts of different dyeing treatments on mechanical strength of hair fibers. Native hair exhibited a strong breaking stress of 223.93 MPa and a high elongation at break of 41.00%. Commercial oxidative treatments severely compromised the hair’s mechanical performance, dropping the tensile stress to 51.39 MPa and the elongation at break of 31.90%. In contrast, hair treated with the SIMNP-based protocol largely avoided this deleterious effect. The SIMNP-dyed hair retained an elongation profile (40.14%) comparable to that of the pristine control, along with a conserved tensile stress of 140.62 MPa. Although the native strength of hair fibers was not fully restored, the SIMNP strategy indeed provided a substantial mechanical advantage over commercial dyes.

#### 3.3.4. Thermal Stability Analysis

**Figure 7:**
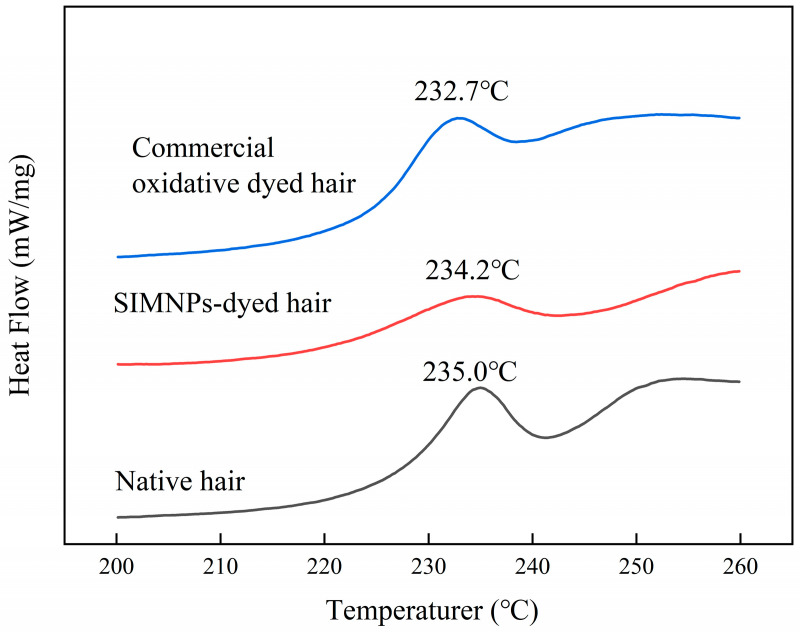
Differential scanning calorimetry (DSC) thermograms of hair treated with different dyes.

DSC was performed to probe the crystalline integrity of the hair keratin matrix (Figure 7). Native hair exhibited a denaturation temperature (Td) of 235.0 °C, reflecting its robust natural cohesiveness. Commercial oxidative treatment caused pronounced destabilization, evidenced by a drop in Td to 232.7 °C. However, SIMNP-treated samples maintained a Td of 234.2 °C, indicating that more native α-helical architecture was preserved compared with the commercial treatment [31].

#### 3.3.5. Photoprotection Against UV Irradiation

**Figure 8:**
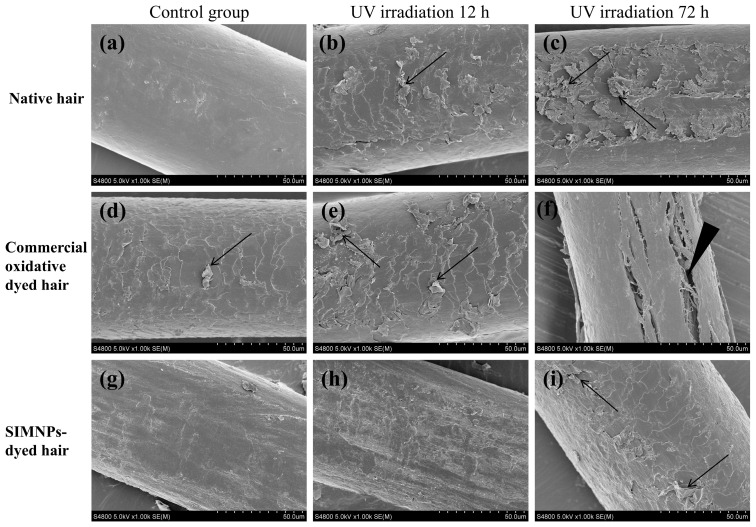
SEM images of hair fibers undergoing UV irradiation. (**a**–**c**) Native hair without UV exposure (control group) and exposed to UV radiation for 12 h and 72 h, respectively. (**d**–**f**) Hair treated with commercial oxidative dye without UV exposure (control group) and exposed to UV radiation for 12 h and 72 h, respectively. (**g**–**i**) SIMNP-dyed hair without UV exposure (control group) and exposed to UV radiation for 12 h and 72 h, respectively. Arrows indicate the lifting, fracturing and peeling of the cuticle, while the black triangle indicates the longitudinal fracture of hair fibers. All images were captured at 500× magnification.

SEM was employed to record the structural evolution of hair fibers under UV stress (Figure 8). The native hair initially displayed its pristine topography with smooth and tightly imbricated cuticles (Figure 8a). However, exposure to UV irradiation induced progressive deterioration. While only marginal scale lifting was observed at 12 h (Figure 8b), prolonged exposure (72 h) precipitated severe structural degradation characterized by extensive curling and significant surface erosion (Figure 8c).

Unfortunately, the hair treated with the commercial oxidative dye exhibited compromised structural integrity even prior to irradiation (Figure 8d). It was found, as shown in Figure 8d–f, that the chemical aggression inherent to the oxidative process resulted in widespread cuticle lifting, localized fracturing and peeling. Such pre-existing damage rendered the fiber highly susceptible to further environmental stress.

Amazingly, the SIMNP-treated fibers maintained a surface morphology comparable to the healthy control before irradiation, confirming the non-invasive nature of the deposition process (Figure 8c). Most importantly, after undergoing rigorous 12 h or 72 h UV exposure, these fibers still exhibited remarkable structural resilience (Figure 8c,f). Apparently, the SIMNP coating was able to effectively prevent hair fibers from undergoing large-scale cuticle unraveling, preserving the overall architecture of the fibers.

### 3.4. Safety of SIMNP-Based Hair Dye

#### 3.4.1. In Vitro Biocompatibility

**Figure 9:**
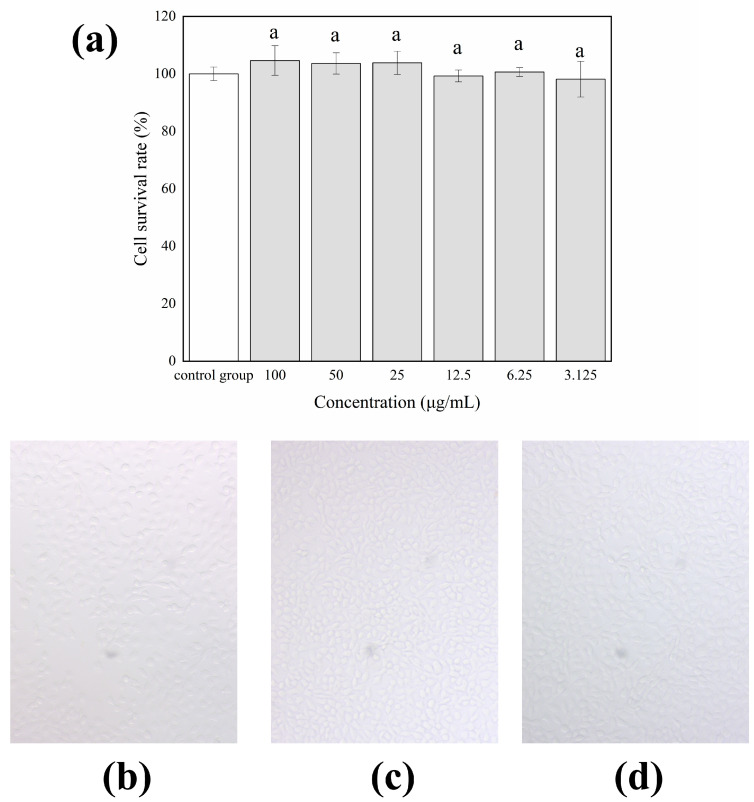
In vitro cytocompatibility evaluation of the formulated SIMNPs. (**a**) Relative cell viability of HaCaT keratinocytes treated with varying concentrations of SIMNPs (3.125–100 μg/mL) for 24 h, as determined by the CCK-8 assay. (**b**–**d**) Representative optical microscopy images of HaCaT cells after 24 h of co-incubation: (**b**) untreated control group (0 μg/mL); (**c**) treated with 100 μg/mL SIMNPs; and (**d**) treated with 3.125 μg/mL SIMNPs. The cells maintained a typical healthy epithelial morphology and high confluence even at the maximum concentration, corroborating the quantitative CCK-8 results and visually confirming the negligible cytotoxicity of the formulation. Different lowercase letters indicate significant differences among the samples.

The biological safety of the dyeing system was evaluated at the cellular level in HaCaT cells using a CCK-8 assay. According to the standard guidelines for the biological evaluation of medical devices and biomaterials (ISO 10993-5), a relative cell viability greater than 70% indicates that a treatment is considered non-cytotoxic [32]. As shown in Figure 9, the relative cell survival rates remained high in the SIMNP-treated cells, fluctuating between approximately 95% and 105% across all tested concentrations (3.125–100 μg/mL). Even at the maximum tested concentration (100 μg/mL), the viability was maintained at 104.6%, and therefore comfortably satisfied the non-toxicity criteria. These results demonstrated that the prepared SIMNPs exerted negligible cytotoxicity and demonstrated favourable in vitro biocompatibility.

Consistent with the quantitative CCK-8 results, optical microscopy observations confirmed that HaCaT cells treated with different concentrations of SIMNPs (3.125–100 μg/mL) maintained a characteristic healthy, cobblestone-like epithelial morphology with robust cell-cell tight junctions and excellent substrate adherence. Notably, no classic morphological hallmarks of cytotoxicity were visibly detected, such as cell rounding, shrinkage, or detachment [32], even at the maximum concentration, indicating that the SIMNPs have good biocompatibility and are suitable for hair dye applications.

#### 3.4.2. Skin Irritation

**Figure 10:**
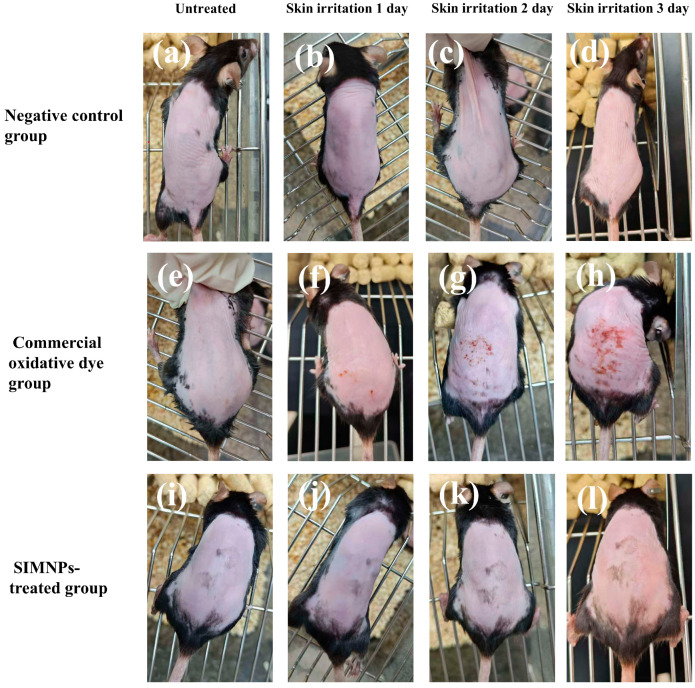
Macroscopic observation of in vivo murine skin irritation. Representative photographs of the depilated dorsal skin of mice subjected to a consecutive 3-day topical application period. Images display the negative control group (physiological saline) at (**a**) untreated baseline and on (**b**) day 1, (**c**) day 2, and (**d**) day 3; the commercial oxidative dye group at (**e**) untreated baseline and on (**f**) day 1, (**g**) day 2, and (**h**) day 3; and the SIMNP-treated group at (**i**) untreated baseline and on (**j**) day 1, (**k**) day 2, and (**l**) day 3. Throughout the observation period, the control and SIMNP groups maintained an intact skin barrier with no visible irritation, whereas the commercial dye group exhibited progressive erythema and eschar formation.

To further assess the practical safety of the dyeing systems, an in vivo murine skin irritation test was conducted, and the results are presented in Figure 10. After 3 days of consecutive topical application, the murine skin treated with the SIMNP mixture showed no observable signs of erythema, edema, or scabbing, exhibiting a healthy macroscopic appearance comparable to the saline-treated control group. Conversely, the skin treated with the commercial oxidative dye at a dose equal to that of the SIMNP formulation developed severe erythema and continuous scabbing within the application area. To quantitatively assess the extent of skin damage, the cutaneous responses were graded according to the standardized Draize scoring system outlined in OECD Test Guideline 404 [28]. The scoring system has clear rules: A score from 0.0 to 0.4 means no irritation. A score from 0.5 to 1.9 means slight irritation. A score from 2.0 to 4.9 means moderate irritation. A score from 5.0 to 8.0 means severe irritation. Consistent with the macroscopic observations, both the physiological saline control and the formulated SIMNP mixture consistently yielded an extremely low Draize score (with a final PII of 0.0 for saline and 0.0 for SIMNPs) for both erythema and edema at all evaluated time points (24, 48, and 72 h after the treatment), confirming a non-irritating classification. In severe contrast, the cutaneous sites subjected to the commercial oxidative hair dye exhibited a rapid escalation in irritation scores (PII), reaching a final PII of 3.11. The progressive development of profound redness and subsequent eschar formation culminated in a maximum erythema score of 4 by the end of the observation period. Collectively, these macroscopic and quantitative evaluations directly demonstrated that the SIMNP-based dye does not induce skin irritation, presenting a significant safety advantage over traditional oxidative formulations.

## 4. Discussion

### 4.1. Bionic Design of the Melanin Nanoparticle-Based Dyeing System

In this study, a bionic hair colorant based on these SIMNPs was developed. It operates through biological mimicry both compositionally and optically. Compositionally, the colorant utilizes natural eumelanin extracted from squid ink. Because this pigment is strictly homologous to the native eumelanin found in human hair, the SIMNP-based strategy might also achieve true optical biomimicry. It could deliver a natural black hue that perfectly matches endogenous hair color, fundamentally differing from the harsh and artificial tones often produced by synthetic aromatic amine dyes [16].

Furthermore, there are some differences between the bionic design of melanin nanoparticles and other biomimetic approaches. Rather than relying on conventional biomimetic approaches that require the harsh in situ oxidative polymerization of synthetic precursors, such as dopamine, to build artificial coatings, the strategy dependent on SIMNPs is able to utilize pre-formed SIMNPs. By employing Fe^3+^ ions to coordinate these intact nano-pigments with hair keratin, this strategy gently anchors the biomaterials onto the hair surface. This process circumvents the destructive chemical pathways of traditional oxidation. This strategy might bring more biosafety and biocompatibility to human individuals, which is also meaningful to bionic design.

### 4.2. Formation and Stability of Melanin Nanoparticles

The microstructure aggregated in crude SIME indicated that endogenous cellular debris, such as proteins and lipids, might act as an interparticle “binder” driving non-specific agglomeration [11,13]. It has been reported that both mechanical disruption and enzymatic hydrolysis can effectively cleave the cementing matrix among squid melanin nanoparticles [33]. In this study, the synergistic action of ultrasound disruption and enzymatic hydrolysis were applied to cleave the proteinaceous matrix and lipid impurities in the raw melanin structure. By stripping away these cementing agents, the process successfully liberated the individual melanin granules from the bulk matrix, yielding discrete nanoparticles (<200 nm). Critically, this nanoscale dimension (<200 nm) was statistically smaller than the typical inter-scale spacing of human hair cuticles [34], theoretically enabling the SIMNPs to bypass the steric hindrance of the cuticle layer and achieve deep cortical penetration, which might be a feat unattainable for SIME aggregates. Additionally, the enhanced surface roughness results in augmented specific surface area, which provides more active sites for subsequent coordination with Fe^3+^ ions [35].

Spectral analyses corroborate this physical dismantling. As shown in the FT-IR spectra of SIMNPs, the complete disappearance of aliphatic C-H stretching peaks (2920–2850 cm^−1^) and a smoother “fingerprint region” (1000–1400 cm^−1^) confirmed the removal of lipid and proteinaceous contaminants [36,37]. Similarly, UV–Vis data confirmed the preservation of the characteristic melanin peak at 216 nm [38], while the absence of 260/280 nm absorption verified the clearance of nucleic acids and proteins [25]. Indeed, the synergistic treatment was helpful in removing the biological matrix without compromising the polyindole–quinone backbone.

The successful removal of these proteinaceous impurities dramatically altered the surface electrochemistry of the melanin particles, particularly in the acidic region. Crude SIME is closely associated with some cationic shielding proteins rich in protonatable amine groups (-NH^3+^), which can counteract the intrinsic negative charge of melanin under acidic conditions [39]. Stripping these proteins might expose high-density latent groups, such as carboxyl (-COOH) and phenolic hydroxyl (Ar-OH), leading to the electronegative enhancement observed in SIMNPs [39]. The exposed anionic sites might also provide abundant anchoring points for subsequent Fe^3+^ coordination [35]. At pH 8.0 (the optimized pH for dyeing, as stated in Section 2.4.3), the SIMNPs demonstrated excellent colloidal stability with a zeta potential of approximately −37.5 mV. According to the Derjaguin–Landau–Verwey–Overbeek (DLVO) theory, the high absolute value of zeta potential found in SIMNPs suggests strong electrostatic repulsive force between these bionanoparticles. This repulsion might create a substantial potential energy barrier that effectively counteracts the attractive van der Waals forces, thereby preventing the flocculation or sedimentation of the SIMNP dispersion [29].

### 4.3. Dyeing Mechanism and Process Optimization

Direct adsorption of SIMNPs onto the hair surface is thermodynamically hindered by strong electrostatic repulsion between the highly anionic nanoparticles (zeta potential of −37.5 mV) and the negatively charged keratin (isoelectric point of 3.7) [40,41]. To overcome this barrier, the pre-mordanting strategy involving a “cationic bridging” mechanism [40] was utilized. The necessity of this bridging is strongly supported by our colorimetric screening: direct dyeing with SIMNPs yielded a lightness L value of 39.69 ± 0.61 and a total color difference ΔE* of 39.47 ± 0.72, whereas the addition of FeCl_3_ significantly improved these values to 16.3 ± 0.63 and 64.13 ± 0.74, respectively. Based on these macroscopic empirical results and the dense nanoparticle deposition presented in Figure 4d, we propose the theory that trivalent ions Fe^3+^ act as electrostatic screening agents to compress the electrical double layer (EDL) of nanoparticles. Serving as in situ nucleation sites, we hypothesize that Fe^3+^ facilitates the formation of a robust keratin–Fe^3+^–melanin ternary complex, linking the amino acid residues of hair keratin with the electron-donating groups (-OH, -NH_2_, -COOH, indole units, amino acid units, etc.) of SIMNPs [42]. This theoretical interaction logically explains the effective anchoring of SIMNPs on hair fibers, as presented in Figure 4c, without the help of traditional cuticle-damaging chemical oxidants [9].

The dyeing efficacy of SIMNPs on the hair was greatly dependent upon Fe(III)-mediated coordination, and process optimization for hair dyeing was necessary. Alkaline conditions (pH 8–10) significantly enhanced dye penetration by inducing the swelling of cuticular scales and reducing steric hindrance, and pH 8.0 was selected to achieve the balance between color depth and fiber structural integrity [40]. Thermodynamically, moderate heating (45 °C) accelerates the diffusion and chelation of nanoparticles, whereas higher temperatures lead to complex instability and desorption. However, it is fully acknowledged that a prolonged treatment at 45 °C for 80 min might exceed the thermal tolerance limit of the human scalp, in the case of real-world clinical or salon use. Physiological studies have established that the thermal pain threshold for human skin is approximately 43 °C, and prolonged exposure at 45 °C significantly increases the risk of thermal injury and user discomfort [43]. Notably, the temperature data (shown in Figure 3d) demonstrated that lowering the temperature to a skin-friendly 40 °C still yielded excellent coloration (ΔE* = 65.91 and L* = 15.13), which was visually indistinguishable from the mathematical optimum coloration at 45 °C (ΔE* = 66.79 and L* = 14.27). Moreover, no statistic difference can be found at the dyeing duration of 80–120 min, while the L* and ΔE* acquired after the 20 min exposure were not far from those found in the 80–120 min groups, suggesting that the dyeing performance is acceptable even with a dyeing time of only 20 min. Thus, less exposure time might be feasible for hair dyeing. Future studies might focus on some alternative optimization solutions to achieve these excellent results at even lower temperatures (e.g., room temperature) and shorter exposure times, which might greatly benefit the application of SIMNP dye for humans. One practical alternative is to incorporate safe cosmetic penetration enhancers (such as urea and liposomes), as these agents can facilitate dye penetration into hair cuticles without thermal assistance [44,45]. Another approach is advanced condition optimization with the help of some statistical tools such as response surface methodology (RSM), which is able to precisely model and balance the trade-off between maximal color performance and practical human comfort.

The behavior of SIMNP colorant during water washing closely mirrored the biphasic washing profile observed in the SIMNP-dyed hair: an initial phase of color loss (cycles 1–7) reflecting the physical desorption of loosely bound, superficial pigment, followed by subsequent stabilization in color. This sustained plateau indicated that Fe(III)-mediated coordination effectively anchored the remaining SIMNPs within the hair fibers. While a minor initial color loss is a common phenomenon for non-oxidative dyes, this coordination-driven method achieves a stable, long-term coloration without the use of cuticle-damaging harsh oxidants [9]. Therefore, it offers a practical foundation for developing marine-based bionic hair dyes. Testing alternative biocompatible metal ions in place of Fe^3+^ could be a logical next step to create a wider range of natural colors while maintaining this low-damage coloring mechanism.

Importantly, this dyeing process clearly articulates how this SIMNP dyeing system advances beyond prior melanin-based or metal–phenolic network (MPN) hair dyes. First of all, unlike conventional biomimetic systems (e.g., polydopamine) that heavily rely on in situ oxidative polymerization under hair-damaging alkaline conditions [15], the SIMNP-based strategy utilizes pre-formed natural melanin nanoparticles, enabling a low-damage and mild anchoring process. In addition, while typical plant-derived MPNs often produce unnatural greenish or brownish shades [41], this squid-ink-derived eumelanin compositionally replicates the native pigment of human hair [16], delivering an authentic deep black color. Furthermore, the inherent insolubility of raw squid ink is innovatively resolved through the green synergistic pre-treatment (ultrasound and enzymatic hydrolysis), guaranteeing the formation of highly uniform nanoparticles that can efficiently penetrate the cuticle gaps prior to Fe^3+^ coordination.

### 4.4. Preservation of Hair Structure and Strength

Tensile strength and thermal stability serve as reliable indicators of fiber health, directly reflecting the stability of the α-keratin helical network and the density of the disulfide cross-links within the hair shaft [46]. The severe structural degradation observed with commercial oxidative dyeing occurs because harsh alkaline swelling and peroxide oxidation indiscriminately cleave the cystine bridges essential to load bearing [33]. In the present study, the preserved fiber integrity was attributed to the non-invasive nature of the SIMNP coloration mechanism. Since the process relies on physical deposition and metal-ion coordination rather than oxidative degradation [46], the SIMNP strategy successfully decouples coloration from structural damage. This macro-level mechanical advantage was deeply substantiated by the micro-level thermal properties reflected by the DSC results.

The denaturation temperature (T_d_) of 235.0 °C observed in native hair reflects its robust natural cohesiveness. Compared with commercial dyeing, the SIMNPs might exhibit superior preservation of the native α-helical architecture [26], as evidenced by a higher T_d_ (234.2 °C vs. 232.7 °C). The thermal data corroborate the favorable mechanical properties. This non-oxidation approach could maintain the vital disulfide architecture easily damaged by conventional dyes, offering a highly protective and environmentally benign paradigm for next-generation hair colorants. Future evaluations could incorporate repeated physical grooming and thermal styling stress tests to further validate the long-term structural benefits of these marine-derived melanin formulations under realistic daily usage conditions.

### 4.5. UV Protection and Photostability

Both UV irradiation and chemical damage can cause unignorable damage to hair fibers. The UV-induced morphological damage in native hair might stem from the cleavage of disulfide bonds and the degradation of the cell membrane complex within hair fibers. This complex acts as the essential intercellular “cement” matrix [47,48] responsible for cuticle cohesion, and then the lifting, fracturing and peeling of cuticle occur as shown in Figure 8b–e,i. Additionally, chemical aggression might exacerbate the inherent vulnerability of hair fibers. The alkaline oxidation commonly applied in commercial dyeing could strip the protective hydrophobic lipid layer containing 18-methyl Eicosanoic Acid (18-MEA), leaving the hydrophilic cortex directly exposed to intense environmental photodamage [43,47]. This would facilitate the development of structural damage to hair, finally resulting in the visible longitudinal cracks on the hair fibers after a 72 h of UV exposure (Figure 8f).

The SIMNPs, however, demonstrated excellent photoprotection, bypassing the structural vulnerabilities mentioned above. The potent protective efficacy might be partly attributed to the high density of melanin coating, which physically shields the underlying keratin matrix form UV irradiation. The broadband photon-sinking capability of the melanin–Fe^3+^ complex can efficiently absorb and scatter high-energy UV photons and dissipate them as heat [49]. Additionally, the inherent radical-scavenging properties of melanin can neutralize surface-bound reactive oxygen species (ROS), which might further mitigate photo-oxidative stress [50]. This implies that the SIMNP-based protocol confers both non-destructive coloration and powerful UV protection. It calls for in vivo evaluations under natural sunlight to further validate the commercial viability of the SIMNP-based dye as a multifunctional hair care product.

### 4.6. Biocompatibility and Safety Evaluation

Beyond structural preservation and UV protection, the clinical translation of hair colorants strictly requires comprehensive safety evaluations. The in vitro assay demonstrated that SIMNPs possess negligible cytotoxicity against HaCaT keratinocytes, maintaining high cell viability (>85%) even at elevated concentrations. This high tolerance is attributed to the inherent biocompatibility of the natural eumelanin polymer [51]. Furthermore, the in vivo murine model provides macroscopic evidence corroborating this safety profile. Commercial oxidative treatments generally need to be operated under harsh alkaline conditions and rely on reactive chemical precursors. In this study, severe skin irritation characterized by erythema and eschar formation on mice skin was found for the commercial dye. In contrast, the SIMNPs caused no such adverse reactions, implying the biosafety of these nanoparticles. By replacing the destructive oxidative pathways with mild, coordination-driven deposition of bio-sourced melanin, the SIMNP-based dyeing system effectively mitigates the risks of chemical burns and contact dermatitis, presenting a highly biocompatible alternative for human cosmetic application.

## 5. Conclusions

In this study, discarded squid ink sacs were used to prepare a bionic hair colorant based on the biological melanin nanoparticles formed in the ink. By synergizing ultrasonic cavitation with enzymatic hydrolysis, the inherent aggregation of crude melanin was overcome, enabling the development of an effective SIMNP-based dyeing system. By replacing destructive alkaline-oxidation pathways with a mild iron mordanting process, this strategy depending on SIMNPs effectively decouples hair coloration from structural damage. The SIMNP-based system delivers a wash-resistant natural black hue while preserving the intrinsic α-keratin architecture of hair fibers and providing robust protection against UV irritation. Crucially, safety evaluations confirmed that this SIMNPs formulation possesses excellent biocompatibility, potently circumventing the severe irritation and allergic risks commonly seen in conventional chemical dyes. While the current thermodynamically optimized dyeing temperature presents a minor limitation on prolonged scalp exposure, future optimization studies can focus on adapting the formulation for milder physiological conditions. To sum up, this study presents a highly biocompatible, low-damage paradigm for the next generation of hair-dyeing agents and establishes a sustainable technological pathway for the high-value valorization of aquatic cephalopod by-products.

## Figures and Tables

**Table 1:** Draize skin irritation scoring system (OECD Guideline 404).

Skin Reaction	Description	Score
Erythema and Eschar Formation	Skin has no erythema.	0
	Skin has very slight erythema. People can barely see the redness.	1
	Skin has well-defined erythema. The red area has clear edges.	2
	Skin has moderate to severe erythema.	3
	Skin has severe erythema. The skin looks beef-red. Or the skin has hard eschar. The eschar covers the skin surface.	4
Edema Formation	Skin has no edema.	0
	Skin has very slight edema. People can barely see the swelling.	1
	Skin has slight edema. The swollen area has clear edges. The skin is raised.	2
	Skin has moderate edema. The skin is raised about 1 mm.	3
	Skin has severe edema. The skin is raised more than 1 mm. The swelling goes beyond the test area.	4

## Data Availability

The original contributions presented in this study are included in the article. Further inquiries can be directed to the corresponding authors.

## Abbreviations

The following abbreviations are used in this manuscript:

DHI	5,6-dihydroxyindole
DHIC	5,6-dihydroxyindole-2-carboxylic acid
DSC	Differential scanning calorimetry
FT-IR	Fourier transform infrared spectroscopy
PPD	p-Phenylenediamine
SEM	Scanning electron microscopy
SIME	Squid ink melanin extract
SIMNP	Squid ink melanin nanoparticle
TEM	Transmission electron microscopy
UV	Ultraviolet
